# SHAP value-based ERP analysis (SHERPA): Increasing the sensitivity of EEG signals with explainable AI methods

**DOI:** 10.3758/s13428-023-02335-7

**Published:** 2024-03-07

**Authors:** Sophia Sylvester, Merle Sagehorn, Thomas Gruber, Martin Atzmueller, Benjamin Schöne

**Affiliations:** 1 Institute of Computer Science, Osnabrück University, Osnabrück, Germany; 2 Department of Mental Health, Norwegian University of Science and Technology, Trondheim, Norway; 3 Institute of Psychology, Osnabrück University, Osnabrück, Germany; 4 German Research Center for Artificial Intelligence (DFKI), Osnabrück, Germany; 5 Department of Psychology, Norwegian University of Science and Technology, Trondheim, Norway

**Keywords:** Explainable AI, ERP analysis, EEG, SHAP, Deep learning, Feature importance

## Abstract

Conventionally, event-related potential (ERP) analysis relies on the researcher to identify the sensors and time points where an effect is expected. However, this approach is prone to bias and may limit the ability to detect unexpected effects or to investigate the full range of the electroencephalography (EEG) signal. Data-driven approaches circumvent this limitation, however, the multiple comparison problem and the statistical correction thereof affect both the sensitivity and specificity of the analysis. In this study, we present SHERPA – a novel approach based on explainable artificial intelligence (XAI) designed to provide the researcher with a straightforward and objective method to find relevant latency ranges and electrodes. SHERPA is comprised of a convolutional neural network (CNN) for classifying the conditions of the experiment and SHapley Additive exPlanations (SHAP) as a post hoc explainer to identify the important temporal and spatial features. A classical EEG face perception experiment is employed to validate the approach by comparing it to the established researcher- and data-driven approaches. Likewise, SHERPA identified an occipital cluster close to the temporal coordinates for the N170 effect expected. Most importantly, SHERPA allows quantifying the relevance of an ERP for a psychological mechanism by calculating an ”importance score”. Hence, SHERPA suggests the presence of a negative selection process at the early and later stages of processing. In conclusion, our new method not only offers an analysis approach suitable in situations with limited prior knowledge of the effect in question but also an increased sensitivity capable of distinguishing neural processes with high precision.

## Introduction

Electroencephalography (EEG) is a powerful tool for studying the neural basis of human cognitive and emotional processing, by offering a non-invasive way to measure the electrical activity of the brain with high temporal resolution. However, the high dimensionality and complex nature of EEG data present significant challenges for data analysis. EEG recordings typically consist of numerous channels with continuous data spanning hundreds of milliseconds, resulting in high-dimensional datasets that are difficult to analyze and interpret. Furthermore, EEG signals are highly variable, with noise and artifacts that can obscure meaningful neural activity. To address these challenges, the first two steps in analyzing EEG data are artifact correction and, most importantly, data reduction.

Two general approaches are commonly used to reduce the dataset to identify time windows and electrodes that index psychological processes of interest and yield significant effects. The first approach is the researcher-driven approach. The researcher chooses or averages across relevant data points and electrodes, constituting an event-related potential (ERP), based on prior knowledge of relevant scientific literature. To this end, the researcher often adapts the predefined time windows and electrode selection based on the data at hand using visual inspection of line plots and topographies. One of the major advantages of the researcher-driven approach is the vast amount of scientific literature available on ERPs and their association to various psychological processes. For example, a positive deflection around 100 ms over posterior electrodes reflects the allocation of attentional resources (P1 component; Hillyard, Vogel, & Luck, 1998). Similarly, time-frequency analysis and their respective time and electrode windows, such as posterior reductions in oscillatory alpha or the mid-frontal theta old/new effect (Lisman & Jensen, 2013), have been extensively researched, providing a clear hypothesis of where to look when researching a particular psychological process.

However, the researcher-driven approach also has several limitations. First, novices may not be aware of all the relevant literature, potentially missing crucial effects. Second, researchers may focus on a specific part of the signal and either miss or create effects, introducing bias into the analysis. Third, when researching new psychological concepts (e.g., semantic self; Schöne, Köster, & Gruber, 2018) or paradigms (e.g., virtual reality; Kothgassner & Felnhofer, 2020; Schöne et al., 2023), it is particularly challenging to come up with an a priori hypothesis about which component (or time-frequency analysis) might yield meaningful results or define a new time/electrode window. To overcome these limitations, a data-driven approach can be utilized to analyze the EEG signal. Multiple permutation tests, especially non-parametric ones, have been shown to be a valuable tool for analyzing EEG data (Maris & Oostenveld, 2007, effectively avoiding the researchers’ bias and assumptions about the data. This approach is especially useful for exploratory analysis by permuting over electrodes and time windows. However, interpreting cluster-based permutation tests can be challenging, especially when significant clusters span multiple time points and electrodes, making it difficult to determine the underlying physiological mechanisms or cognitive processes contributing to the observed effects, especially when the size and shape of the resulting clusters may be difficult to interpret. The precision of the temporal, spatial and frequency extent is easily overestimated, leading to unwarranted conclusions about the on- and offset and electrode distribution of specific effects (Sassenhagen & Draschkow 2019. Researchers may furthermore need to rely on additional visualization techniques or statistical tests to understand the clusters’ significance fully. For example, source localization aids the interpretation of the results of the cluster-based permutation test.

Most importantly, the cluster-forming thresholds, i.e., the minimum number of electrodes that form a cluster and the adjunct time points, can be arbitrary as there is no universally accepted rule. Hence, the distribution of the data, or visual inspection, is used to determine a threshold. A threshold set too high may lead to increased specificity but reduced sensitivity. In contrast, a low threshold may increase sensitivity but reduce specificity. Again, this may introduce bias and lead to inconsistent results across studies. The theoretical foundation for forming clusters is that brain activity is spatially and temporally contiguous, so data at neighboring electrodes are highly correlated as they index the same psychological process. Clustering data does not only make it easier to interpret the results in terms of the underlying neural processes, but also has statistical benefits. The more electrodes form a cluster, the more likely artifacts are to be averaged out. Furthermore, it reduces the multiple comparison problem that arises when analyzing large numbers of electrodes and time points.

To overcome the limitations of the two approaches mentioned above while leveraging their affordances, we developed a method that incorporates a convolutional neural network (CNN) (Li, Liu, Yang, Peng, & Zhou Li et al. (2021)) and SHAP (SHapley Additive exPlanations) (Lundberg & Lee, 2017) as an explainability method to automatically and objectively identify the most relevant time points and electrodes in ERP analysis. SHAP-driven ERP analysis (SHERPA) provides a comprehensive and data-driven approach to identifying meaningful patterns in ERP data by utilizing the high spatial resolution of EEG, allowing for the identification of smaller clusters or even individual electrodes. By focusing on the most relevant features, i.e., electrodes at specific time points, this approach enables the identification of more specific and localized patterns of the underlying neural processes, which could lead to a better understanding of the cognitive and neural mechanisms underlying various mental processes.

In order to demonstrate the feasibility of our approach, we choose a typical ERP component in research on human perception - the N170. It represents the well-established electrophysiological correlate of human face perception (Rossion & Jacques, 2011). The N170 is a characteristic stimulus-dependent deflection with negative amplitude that occurs between 130 and 200 ms, peaking at about 170 ms after presentation of a picture of a face (Itier & Taylor, 2004; Rossion & Jacques, 2011). The N170 exhibits robust and reproducible face-sensitive properties and thus provides an electrophysiological index for face perception (Eimer, 2011). Stronger amplitude deflections occur for faces compared to objects (e.g., houses, flowers, cars, etc.; Bentin et al., 2007; Itier & Taylor, 2004), for emotional facial expressions compared to neutral ones (Blau, Maurer, Tottenham, & McCandliss, 2007), as well as for expression changes (Miyoshi, Katayama, & Morotomi, 2004). The particular shape of the N170 in response to faces, referred to as the face effect, is well known and has been frequently reproduced in the scientific literature on face perception (Rossion & Jacques, 2011). The topographical distribution of the N170 covers occipital-temporal sites, i.e., posterior visual cortical areas, with maximal amplitude deflections at lateral posterior electrodes (i.e., P8/P7, PO8/PO7; Eimer, 2011; Rossion & Jacques, 2008, 2011). However, the face effect diminishes when posterior midline electrodes (O1, O2, Oz) are included in the EEG analysis, hence those are usually not used (Rossion & Jacques, 2008).

In addition to objects, the perception of faces is often compared to control images that differ at the configural and/or featural level, i.e., the facial stimuli are blurred or scrambled (e.g., Bombari et al., 2013; Civile et al., 2018; Herrmann, Ehlis, Ellgring, & Fallgatter, 2005; Rossion, 2014; Schwaninger, Lobmaier, Wallraven, & Collishaw, 2009; Zion-Golumbic & Bentin, 2007). Although a standard procedure, the results comparing faces with different forms of perceptual control images do not yield a clear picture in terms of N170s face sensitivity. Blurred faces generally evoke weaker N170 responses (e.g., Rossion, 2014) but scrambled faces can either also elicit weaker deflections (e.g., Civile et al., 2018) or stronger deflections (e.g., Zion-Golumbic & Bentin, 2007) compared to normal faces.

Considering the long history of the N170 and its pivotal role in face perception research, it is a standard electrophysiological measurement, making it ideal to validate new approaches to data analysis.

The main contributions of this paper are as follows:

- We train a (DL-based) classifier to see if the three conditions (normal faces vs. blurred faces vs. scrambled faces) of a previously conducted experiment (Sagehorn et al., 2023) can be distinguished objectively and reliably using EEG data only. Our approach utilized data obtained through standard EEG methods, including acquisition and preprocessing, demonstrating its compatibility with existing data and ease of application.
- We apply an explainable artificial intelligence (XAI) method, specifically SHAP, to identify important spatial and temporal coordinates which contribute most to the classification and hence are the most important to distinguish the conditions.
- Consequently, these coordinates can facilitate the discovery of novel EEG components or validate the existence of previously identified components in an objective and empirical manner.
- We directly compare this new DL-based approach (SHAP-driven ERP: SHERPA) with conventional approaches to pinpoint affordances and limitations of each method.

The remainder of the paper is organized as follows: “Background” summarizes the most important machine learning (ML) and DL concepts. After that, “Related work: machine learning in EEG analysis” discusses relevant literature. Next, the “Methods” section provides an overview of the data, XAI pipeline, and conventional analysis methods used in this study. In the “Results” section, we present the outcomes of these different methods, and in the “Discussion” section, we critically reflect on them. Subsequently, we offer detailed guidance on reporting (“Reporting SHERPA results”) and interpreting (“SHERPA interpretation”) the findings. Following that, we present the “Limitations” of the approach and, lastly, draw a general “Conclusion”.

## Background

Classification is the operation of mapping instances, in our case trials, into target classes, here the experimental conditions. In supervised classification this mapping is based on a known relationship between characteristics, i.e., features, of instances and known class assignments, which are generalized (Nisbet, Miner, & Yale, 2018). Machine learning is an umbrella term used for mathematical algorithms trained to solve a single task; they should provide an estimate for a solution without being explicitly provided with a way to solve the problem. In the standard case, the algorithms learn on a training dataset and in a second step it is tested how good the resulting models generalize to unseen cases using a testing dataset. Hence, for supervised classification tasks, a training set with instances and their features as well as their target classes is provided, so that the model can “learn” the relationship between the provided features and the target class. A great variety of algorithms for such tasks exists, among them artificial neural network (ANNs). ANNs are essentially a mathematical model similar to the structure of a brain. Many small units called neurons connected to each other and structured into layers perform a series of linear and non-linear operations on the input data to derive at the desired outcome. In the case of supervised classification, the desired outcome is the target class associated with the instance. Particularly large architectures including many layers and units with often complex connections are subsumed under the term DL. In its simplest form, a series of operations within the neural networks is performed in one direction only (feed forward neural network). However, many more complex architectures were created, e.g., Hosseini, Hosseini, and Ahi (2021) and Krogh (2008). For a comprehensive overview on artificial neural networks, see e.g., Krogh (2008).

A CNN is a deep feed forward neural network. The modules comprising convolution layers and pooling layers are inspired by the visual cortex, with its alternation of simple and complex neurons. The convolutional layers serve as feature extractors as the neurons with their receptive fields are arranged into feature maps. The pooling layers are used to reduce the spatial resolution of these feature maps. Max pooling, as used here, preserves the maximum value within a receptive field only. The dense layers that follow interpret the features and provide the high-level reasoning, i.e., in this case the classification. For a comprehensive overview, see Rawat and Wang (2017).

Explainable artificial intelligence (XAI) (e.g., Atzmueller & Roth-Berghofer, 2011; Barredo Arrieta et al., 2020; Biran & Cotton, 2017; Gunning, 2017) is a field complementing classical ML and DL research and, most importantly, aiming to provide explanations for the results presented by these models. The goal is to enhance trust in the model by giving reasons for the predictions and decisions that the model provides. While in ML intrinsically interpretable models like tree structures and small regression tasks are present, DL models are inherently black-boxes, i.e., not understandable by and interpretable for humans. In most cases, intrinsically interpretable models are incapable of reaching a sufficiently high performance, black-box models on the other hand lack the interpretability necessary to trust the model. (Molnar, 2022)

Despite progress in making deep learning models more interpretable, the degree of interpretability achieved varies widely, especially for complex tasks such as ERP analysis. While progress has been made in making deep learning networks intrinsically interpretable, full transparency across all layers remains a challenge. Considering the focus of our study on ERP analysis, where interpretability in the entire original feature space is critical, partially interpretable features are insufficient. We require a method that extends interpretability beyond selected layers or components to the entire feature space. This requirement has led us to adopt post hoc explainers, which, despite advances in interpretable deep learning, are more closely aligned with our need for comprehensive interpretability in the context of EEG analysis.

One of the most frequently used post hoc explainability methods is SHAP, which is employed to compute feature importance. SHAP values are based on Shapley values from cooperative game theory and calculated by individually introducing each feature into a conditional expectation function of the output of the model and then attributing the change produced at each step to the feature newly introduced, while finally averaging the process over all possible feature orderings. Post hoc explainers like SHAP use access to the original model, either in the form of queries (black box) or in a direct form (white box), e.g., using the gradients, to train an explanation model. The SHAP gradient explainer we used in this study is a white box explainer, i.e., it has access to the original model and uses the gradients to calculate feature importance. (Lundberg & Lee, 2017; Molnar, 2022)

In this study, SHAP was chosen because of its established effectiveness in XAI, as evidenced by its widespread citation, particularly in Lundberg and Lee’s seminal paper (Lundberg & Lee, 2017). As SHAP has also demonstrated proficiency in time-series analysis (Alsuradi, Park, & Eid, 2020; Theissler, Spinnato, Schlegel, & Guidotti, 2022) it provides a methodologically sound approach capable of navigating the complexities inherent to the data at hand. Most importantly, our study requires post hoc explanations that provide global insights into feature importance. SHAP’s ability to provide a comprehensive view of critical features in time series data intuitively interpretable for experimental psychologists and neuroscientists makes it a good fit for an application in this field.

## Related work: Machine learning in EEG analysis

While there has been a considerable amount of research conducted in the field of machine learning for EEG data analysis in various applications, foremost in the area of brain–computer interface (BCI), ERP detection remains a niche area of research. Despite this, several relevant studies have been conducted in this area. In this section, we provide an overview of these.

Akhter et al. (2020) presented a study that aimed to extract features to detect the P300, a typical ERP component, from EEG data acquired in an oddball paradigm. The authors calculated five features, such as the maximum amplitude, from 2 s epochs and used them for classification.

Li et al. (2018) and Li et al. (2019) proposed two approaches based on restricted Boltzmann machines (RBMs) for P300 detection in a spelling task in BCI. In their first study (Li et al., 2018), they used a multichannel temporal restricted Boltzmann machine (MTRBM), which was later revised (Li et al., 2019) to a spatial-temporal discriminative restricted Boltzmann machine (DRBM). Both approaches were tested on BCI benchmark datasets and a custom data set for spelling, and achieved state-of-the-art performance.

Havaei et al. (2023) introduced a hybrid approach for P300 detection, combining Gabor transform (GT), modified histogram of oriented gradients (MHOG), and a convolutional neural network (CNN) on spectrograms. The GT and MHOG provide complementary patterns that are interpreted by the CNN, resulting in a smaller and faster approach compared to a traditional CNN. The approach was tested on BCI benchmark datasets and achieved high accuracy in detecting the P300.

Lan et al. (2021) proposed a Multi-Attention Convolutional Recurrent Model (MACRO), which combines multi-attention with a recurrent neural network (RNN) and a CNN for P300 detection. The CNN was used for spatial-temporal feature extraction, and the multi-attention head was used to identify discriminative channels and temporal periods in the signal. The approach was tested on a BCI benchmark dataset with minimal preprocessing and outperformed the state-of-the-art while showing good generalization to unseen subjects.

Santamaría-Vázquez et al. (2020) proposed EEG inception for BCI in a spelling task similar to the one in Li et al. (2019). The authors used a CNN to extract complex features and combined it with inception, resulting in a lighter approach that outperformed other state-of-the-art approaches in detecting the P300.

The majority of the studies discussed in this section have been centered around the detection of the P300 event-related potential. Although these studies have yielded promising results, it is clear that there is a significant lack of research on other ERP components, such as the P100 or N170. The absence of this research raises questions as to whether the strong outcomes found in P300 studies can be extrapolated to other components. Furthermore, the N170 is believed to reflect distinct cognitive processes (i.e., attentional gain to certain objects), whereas the P300 is a rather unspecific component which indexes several neuronal processes. Hence, the N170 is ideally suited to validate our new approach.

Most importantly, the approaches presented above are not designed to discover a new ERP component, but rather show a way to detect a known one, assuming prior knowledge of its location and timing. The supervised classification parts are usually designed to predict the presence or absence of a specified component, and hence achieve very high accuracies in this binary classification.

In contrast, we used the experimental conditions of a psychological experiment on face perception as targets for our classifier. The experiment consisted of three conditions: faces, blurred faces, and scrambled faces. The latter two were used as control conditions, designed to elicit no or a weaker N170 as compared to the face condition. Thus, the labels in the experiment implicitly reflect the presence or absence of the N170, though it is not the target directly. Our goal is to detect the N170 component, specifically its temporal and spatial coordinates, using XAI methods without incorporating prior knowledge.

As XAI is a relatively new field, the number of studies using it to explain their results on EEG data is limited. Islam et al. (2022) aimed to predict strokes using a boosting classifier after careful feature extraction. LIME and ELI5, which are both post hoc local explainers (Agarwal & Das, 2020), i.e., focusing on the explanation of single instances without giving an overall account for the entire dataset, were employed. The authors found that the explainability methods largely agreed and aligned with domain expertise in identifying features important to the prediction.

Morabito et al. (2023) employed a combination of CNN and the GradCAM method (Selvaraju et al., 2017) to create channel-frequency maps for the supervised classification of mild-cognitive impairment (MCI) versus Alzheimer’s in EEG data. Even though their dataset included only four patients diagnosed with MCI at the first testing and then with Alzheimer’s at the second, they were able to identify important channels using GradCAM.

Ellis et al. (2022) aimed to detect biomarkers of schizophrenia using a random forest and a support vector machine as classifiers, combined with permutation feature importance. As the classifiers are not capable of dealing with time series data, the authors extracted spectral features in addition to the channels beforehand and performed the permutation separately on both. The two groups of features produced differing explanations, but both aligned with existing literature on schizophrenia biomarkers.

Alsuradi et al. (2020) trained two Extreme Gradient Boosting (XGBoost) (Chen and Guestrin, 2016) classifiers followed by Shapley values on the beta band power spectral densities (PSD) in an active touch task distinguishing trials with and without haptic feedback. The first classifier aimed to identify the most influential channel using a combination of peak value and latency, while the second was trained directly on beta band PSD data of the identified channel to determine the most important time period. The study found that a frontal electrode was the most important channel and that the peak after approximately 700 ms, more pronounced in trials with haptic feedback, was the most important time period. These results align with previous studies and confirm the importance of the beta band for such tasks, as well as the ability of SHAP to deal with highly correlated features.

While there are a few approaches combining ML or DL with XAI and in the case of the last mentioned study (Alsuradi et al., 2020) even findings from typical conventional EEG analyses could be corroborated, to the best of our knowledge, we are the first to use this combination of methods to detect ERP components objectively.

## Methods

### Data

**Fig. 1:**
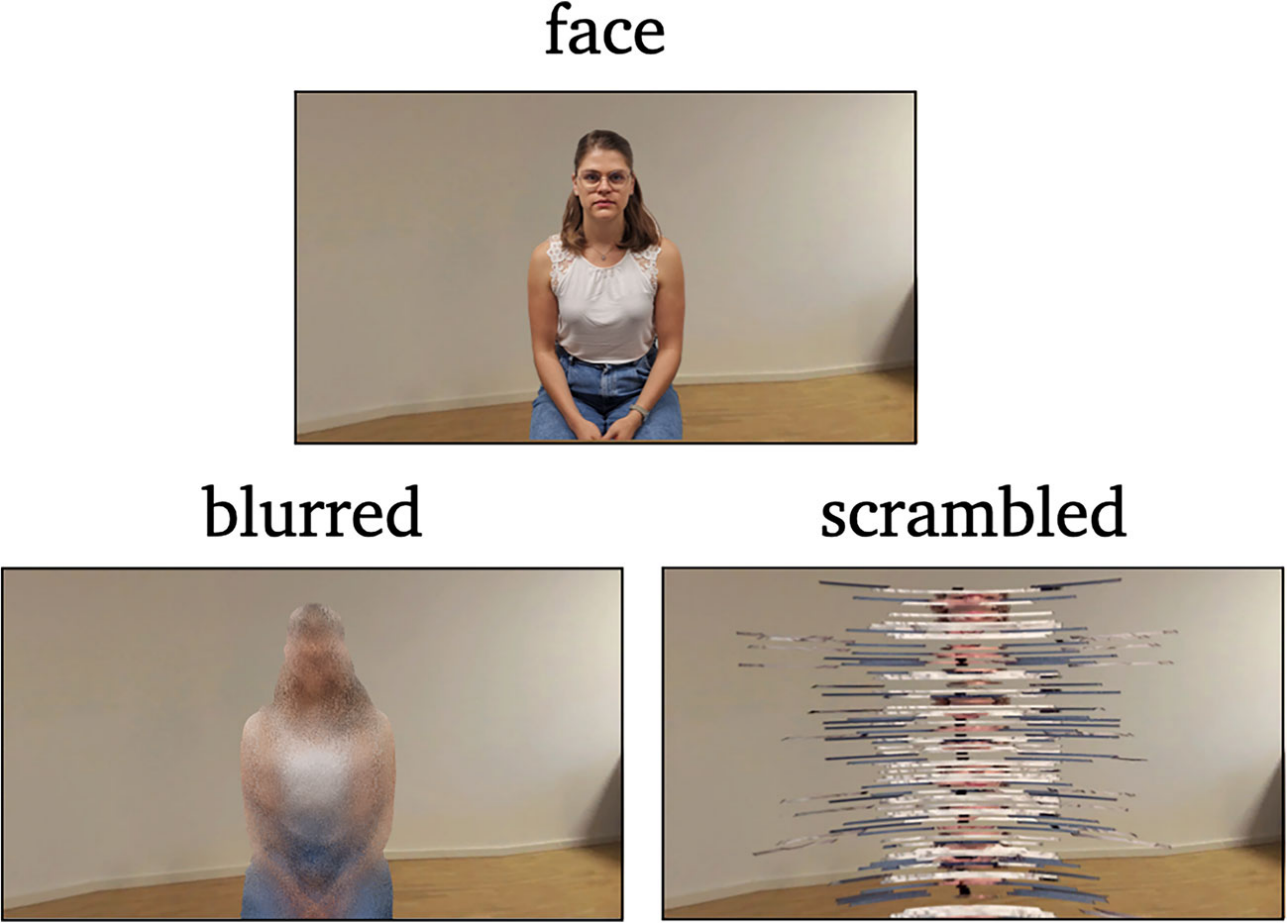
The stimuli. In the experimental condition, the participants saw a normal face (*top*), and in the control conditions either a blurred (*left*) or scrambled (*right*) face

The data used for the present analyses were recorded during a study by Sagehorn et al. (2023) employing a standard paradigm to study face perception. In the part of the experiment relevant to this study, participants sat in front of a computer screen and passively observed pictures of either faces, blurred faces, or scrambled faces (Fig. 1). The latter two comprise the control conditions, while the former is the target. During the experiment, EEG was recorded with 128 electrodes attached according to the international 10-20 system. With the N170, the expected ERP was found using typical EEG analysis.

Twenty-six participants took part in the experiment and completed 60 trials per condition. The data were re-referenced to average reference, high-pass filtered at 0.25 Hz and low-pass filtered at 30 Hz. Subsequently, the data was corrected for artifacts using an independent component analysis and segmented in time-windows from –500 ms to 1500 ms relative to stimulus onset. A baseline correction from –300 ms to 0 ms was applied. The data processing corresponds to the standard procedure for any conventional analyses, hence for further details we refer the reader to Sagehorn et al. (2023). For further processing with SHERPA the data was reduced to 0 ms to 1500 ms.

**Fig. 2:**
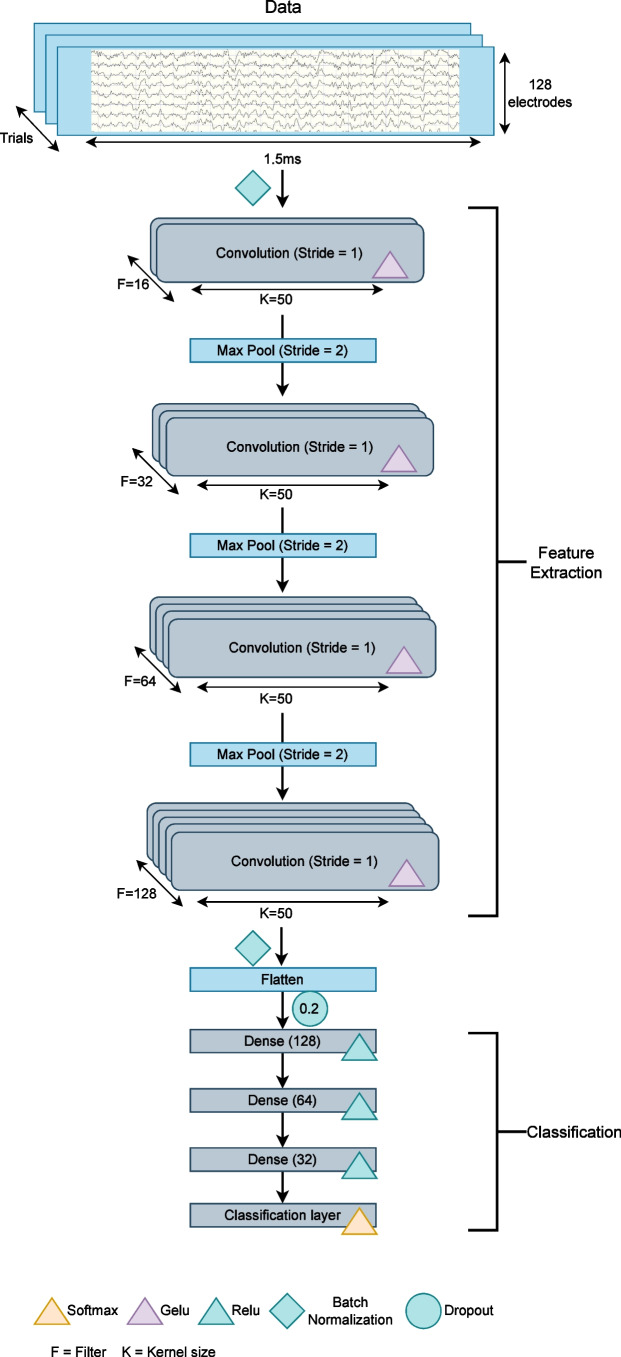
The classification model. The model is composed of four convolutional layers for feature extraction and four dense layers for classification

### Classification and validation

A CNN classifier with four convolutional layers alternating with Max Pooling layers, and Batch Normalization in the beginning and end was used for training. Furthermore, after flattening, four dense layers were added. The convolutional layers have ascending filter sizes between 16 and 128 filters, a kernel size of 50, Gaussian error linear unit (GELU) activation, and L2 regularization. The first convolutional layer employs a GlorotNormal kernel initializer. Dense layers consist of 32 to 128 neurons (descending in number) with rectified Linear Unit (ReLU) activation. The output layer is a dense layer with the number of neurons corresponding to the number of target classes, i.e., three, and softmax activation (Fig. 2).

We used Adam as optimizer for the categorical cross entropy loss. For training, a batch size of 64 and 200 epochs with early stopping after 50 epochs without improvement in validation loss were employed. Before training, we created a hold-out test set from the data and used five-fold cross validation during training for validation. In the context of our study’s focus on a fundamental attention mechanism, expected to be consistent across individuals, we designed our data splitting process to account for potential inter-individual variance. Preliminary tests confirmed that such variance does not significantly influence our results. Therefore, for the cross-validation, we shuffled the data prior to splitting into folds, ensuring no bias from the sequence of data. The holdout test set was composed of a mix of trials, including both participants previously encountered and not encountered by the model during training, providing a robust test of the model’s performance. The weights from the epoch with the best validation accuracy in every fold were then used for prediction on the test set and averaged to get the final estimate of loss and accuracy. The CNN was implemented using TensorFlow^1^, and the cross validation using Scikit-learn^2^.

We validated our approach by means of accuracy, as the dataset is perfectly balanced without missing data and hence no bias could be introduced by unbalanced samples. We chose a CNN, as it performed well in other ERP detection studies (Craik, He, & Contreras-Vidal, 2019). Surprisingly, the survey found that CNNs performed well on the signal directly without the need to transform the data to spectrograms during preprocessing (Craik et al., 2019). Hence, we use the cleaned signal after separating it into epochs as input for the classifier. We also oriented the choice of hyperparameters on existing literature but in the end the results of the manual hyperparameter tuning were decisive.

### Explainable AI

The SHAP gradient explainer^3^ was employed as post hoc explainer in order to get insights into the importance of features, i.e., electrodes and time points. As the explainer is not optimized for time series data, the output consisted of one explanation per time point in our data (i.e., 768 explanations). Thus, we aggregated the absolute data over trials and target classes and plotted the resulting $time \times electrodes$ matrix using a contour plot in order to visually identify clusters of relevant time points and electrodes. In order to formalize this visual inspection, we summed up the SHAP values for one time point using all electrodes and extracted the most important time points. To this end, we used a SciPy^4^ function designed to find local extrema by comparing neighboring points and returning the most extreme in the time window, where the window size is a hyperparameter. Since we took the absolute values of the SHAP values, we were only interested in the local maxima, giving us the most important time points. Starting with the highest local maximum, we then cut out a window around it using the 2D matrix, aggregated the values over the window and hence had the most important electrodes for that time window. The length of the window can be determined by a hyperparameter and was set to 40 ms in this study.

### Cluster-based permutation analysis

To evaluate the eligibility of the electrodes and the time-window that SHERPA produced, a cluster-based permutation analysis of the EEG data is performed. It provides a solution for the multiple comparison problem that is particularly present in data-driven EEG analysis and thus is progressively used in EEG research (see e.g., Tagliabue et al., 2019; Urigüen, Garcia-Zapirain, Artieda, Iriarte, & Valencia, 2017). In cluster-based permutation tests firstly a test statistic is computed for every electrode and time point and then neighboring ones are clustered applying a predefined threshold. Permutations are computed on the clusters thereby decreasing the number of tests and hence the risk of false positives. However, spatial resolution is sacrificed in the process. Furthermore, the clustering threshold greatly affects sensitivity and specificity of the analysis. In the absence of suitable guidelines for the choice of the threshold, the analysis, while data driven, still includes an arbitrarily chosen hyperparameter (Meyer, Lamers, Kayhan, Hunnius, & Oostenveld, 2021). Most importantly, cluster-based permutation tests do not allow drawing inferences on the spatiotemporal location of the effect as the false-alarm rate is not controlled for time and/or channel (Sassenhagen & Draschkow, 2019).

In our experiment, three stimulus types are compared in a within-subject design. Hence, the dependent F-statistic is used to calculate the effect at sample level with a sample specific critical value ($p < 0.05$) as a threshold for clustering. The selected electrodes are clustered on the basis of temporal and spatial adjacency, the latter having been manually determined beforehand for the channel layout of the obtained EEG data (BioSemi ABC Layout with 128 electrodes^5^). The minimum number of neighboring channels for clustering is set to two channels. The maximum sum of the cluster-level statistics (i.e., sum of the sample-specific F-statistics that belong to a specific cluster) is used as the test statistic to be evaluated under the permutation distribution. The significance probability is calculated using the Monte Carlo method with a critical $\alpha$-level of $\alpha =0.05$ for the permutation test and 500 randomizations (i.e., number of draws from the permutation distribution).

In order to compare the number and distribution of electrodes indicating an effect of stimulus type between SHERPA and the cluster-based permutation analysis, any significant clusters resulting from the permutation analysis are considered in the early time window generated by SHERPA (136.25–176.25 ms) (see “Explanations”).

### Conventional researcher-driven analysis

Moreover, the results from the CNN are compared to results obtained from a conventional researcher-driven analysis. Based on prior literature on face perception, relevant electrode positions are derived (Rossion & Jacques, 2008; Boehm, Dering, & Thierry, 2011; Latinus & Taylor, 2006; Dering, Martin, Moro, Pegna, & Thierry, 2011). By visual inspection of the ERP at these electrode positions, the time window for the component of interest (e.g., the N170 component) can be determined. Next, the topography over the chosen time window is inspected to confirm and, if necessary, adjust the electrode positions ultimately used for statistical analysis. This approach is henceforth referred to as the “classical approach”.

The mean N170 amplitudes for all stimulus types are extracted and tested against each other in a one-way repeated measurement ANOVA (rmANOVA) with the factor *stimulus type* (Face vs. Blurred vs. Scrambled). Specifically, the amplitudes for the N170 are extracted for the literature-based electrodes (i.a., P8/P7, PO8/PO7, P10/P9) and a manually defined time-window (165 - 205 ms).

## Results

### Classification

**Fig. 3:**
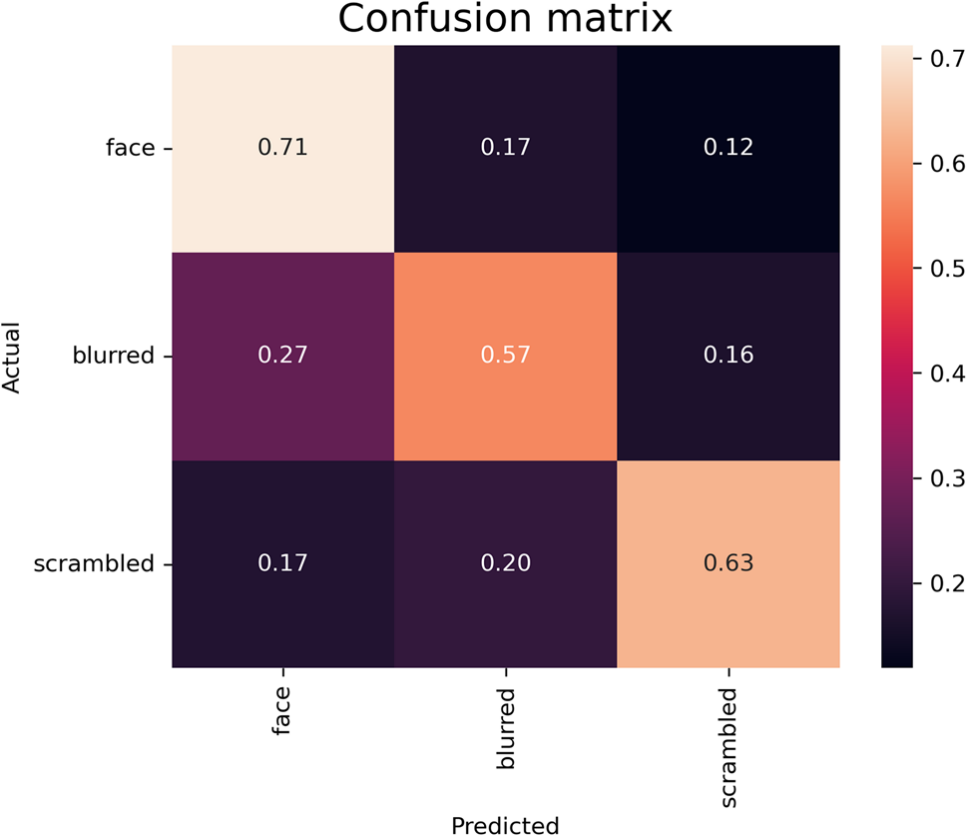
Confusion matrix. Relative frequencies of hits and misses. The *x*-axis shows the predicted target class and the *y*-axis shows the actual target class. All hits are shown in the diagonal

The classifier performed on average with 64% accuracy on the test set. The standard deviation was 0.025. The confusion matrix (Fig. 3) is based on the training of the last fold as an example, and shows that the model detected faces more reliably than the blurred or scrambled stimuli. There, the model confused blurred stimuli more often with faces than scrambled stimuli, indicating that the blurred stimuli might be the more complex distractor. Overall, the performance of the model is nearly two times higher than chance level.

### Explanations

**Fig. 4:**
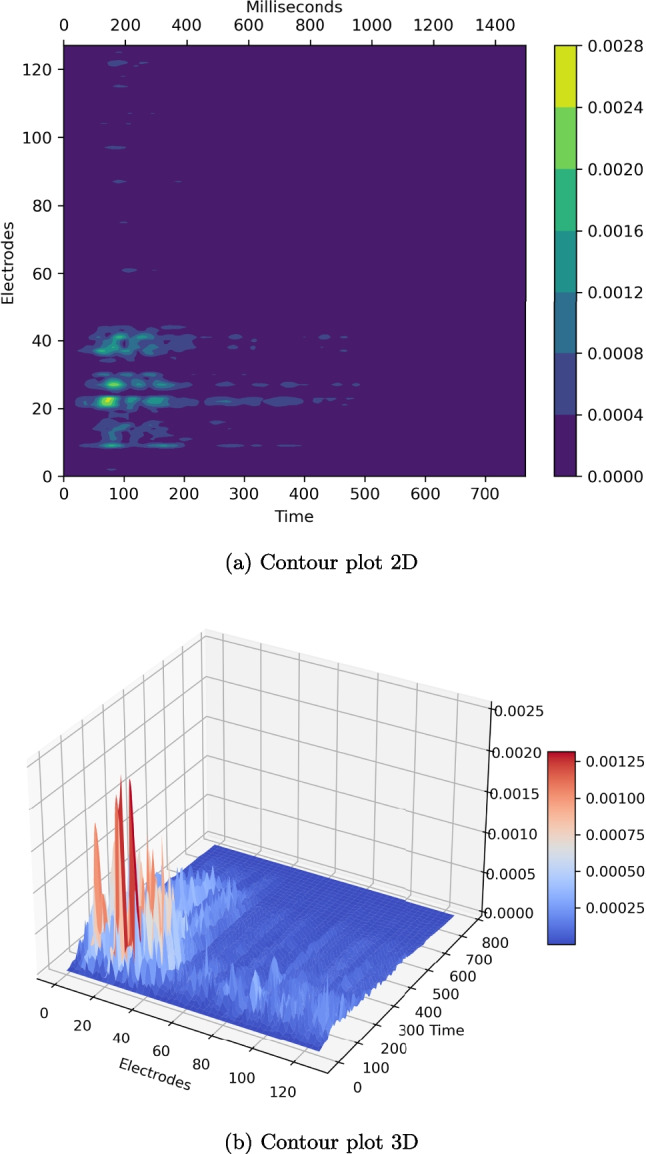
Contour plots. All SHAP values for each electrode at each time point are shown. In (**a**) color and in (**b**) color and amplitude represent the SHAP values. A pronounced cluster of important electrodes at occipital locations and happening early (before 200 ms) is visible in both plots. Importance is indicated by high SHAP values

Aggregating the results of the SHAP explanations over the trials and target classes in contour plots (Fig. 4), it becomes readily visible that there are clusters of important times and electrodes that contribute most to the classification. Visual inspection of the plot shows that the highest SHAP values cluster at occipital electrodes (number from 2 to 30) and an early time window, i.e., before 200 ms.

**Fig. 5:**
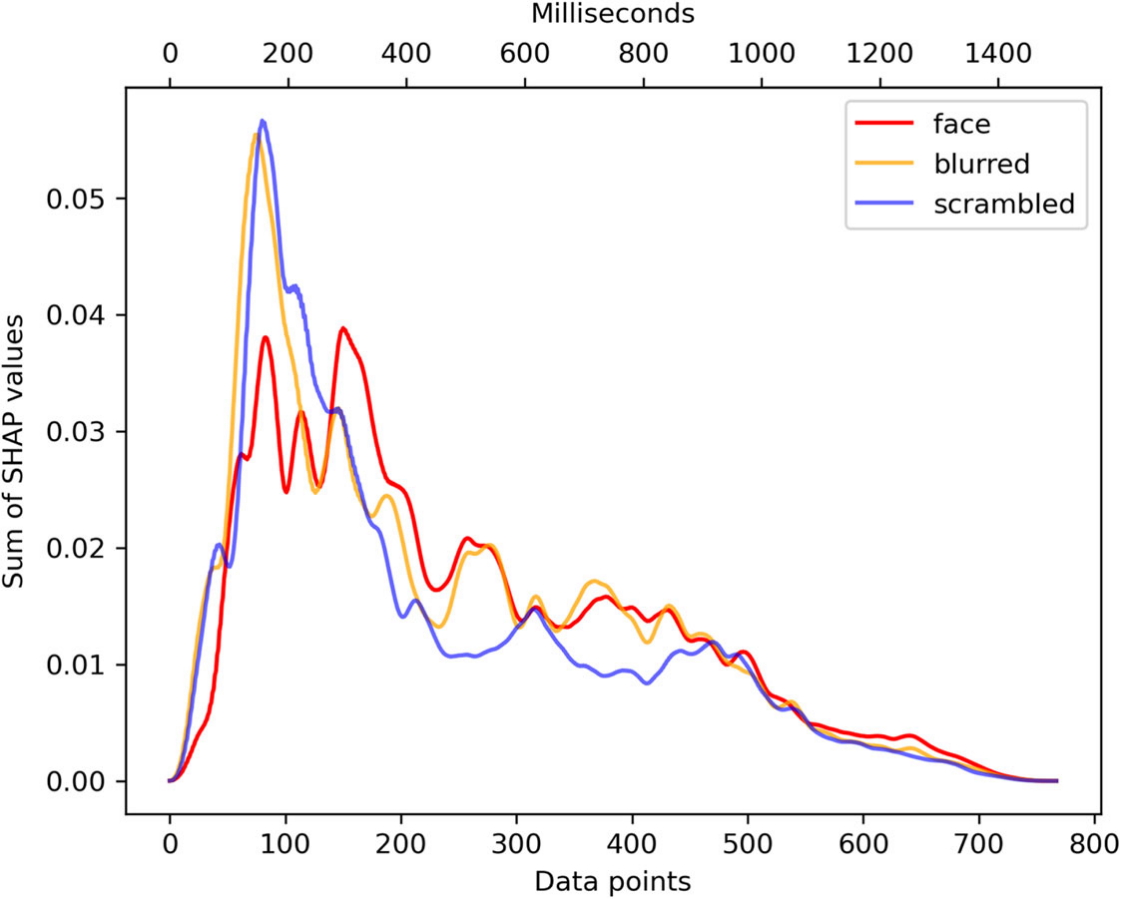
Line plots for the three target classes. Each *line* shows the SHAP value for each time point summed over all electrodes. The *peaks* indicate the most important time points for each target class

**Fig. 6:**
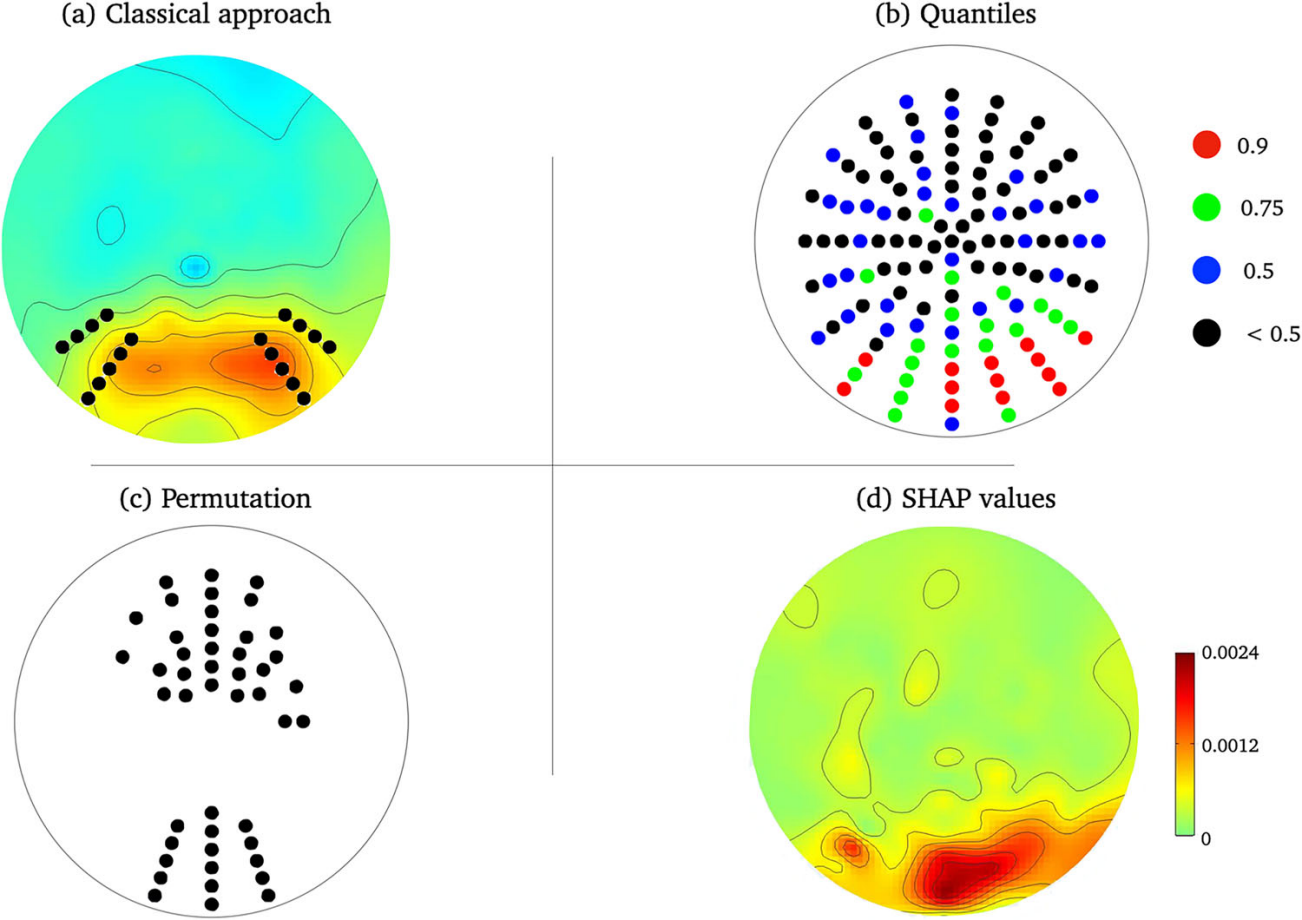
Topographies summarizing the results of the first peak. **a** Topography averaged over all conditions with electrodes analyzed for classic research-driven approach. **b** Quantiles for the SHAP values. **c** Significant cluster from the cluster-based permutation test. **d** SHAP values indicating the most important electrodes for differentiating between the conditions

We defined the most important time windows separately for the target classes (Fig. 5). While the pattern looked similar for scrambled and blurred stimuli with a peak roughly around 150 ms (156.25 and 148.44 ms, respectively) and a summed SHAP value of roughly 0.056 (0.0566 and 0.0555, respectively), the pattern for face stimuli differed considerably. It shows two pronounced peaks at 162.11 and 292.97 ms, both reaching a summed SHAP value of nearly 0.04 with the later peak being a little higher (0.0388) than the former (0.0381). At the later peak as defined by the face condition, blurred and scrambled faces did not exhibit a local maximum (both 0.0313).

**Fig. 7:**
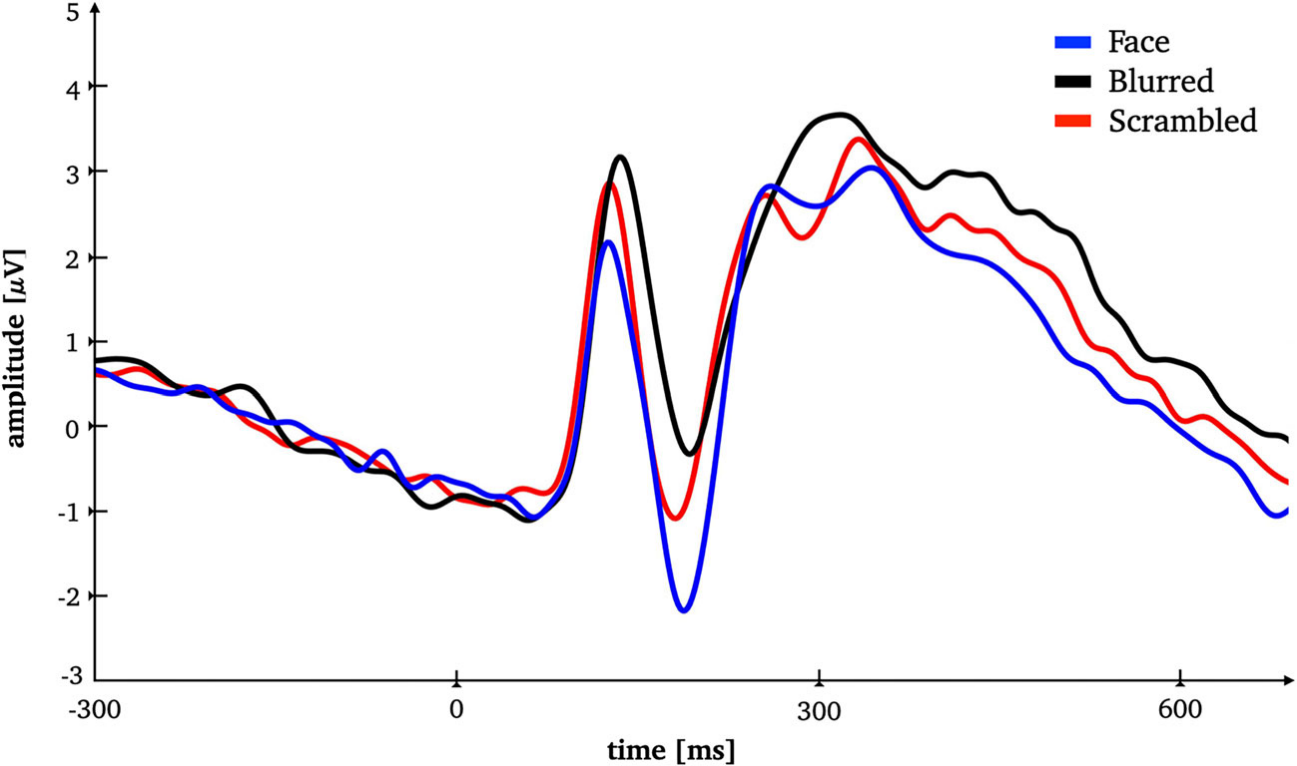
Lineplot for most important electrodes. ERP lineplot for the 0.9 and 0.75 quantile electrodes

For each of the four mentioned peaks (two for faces, and one each for blurred and scrambled stimuli), we created a time window and analyzed which electrodes contributed most to the classification. For this study, we arbitrarily chose a window of 40ms, with the peak being the exact center. For all four time windows, we found occipital electrodes to be the most important, as was already suspected from the contour plots. All three stimulus types show a high agreement. The top three electrodes are always centered and further electrodes in the 90th percentile centered and on the right side, with only very few electrodes on the left side (Fig. 6). Furthermore, calculating the ERPs on basis of the most important electrodes identified by SHERPA, reveals the typical waveform (Fig. 7).

### Conventional analysis approach and cluster-based permutation

The rmANOVA for the classical approach revealed a strong significant effect (*F*(2,50)=5.49, *p*=.007, $\eta ^{2}$ =.180). Blurred faces yielded a significantly weaker N170 deflection than normal faces (*t*(25)=-3.46, *p*=.002) and scrambled faces (*t*(25)=-3.14, *p*=.004). The N170 amplitudes for normal and scrambled faces were not significantly different (*t*(25)=.006, *p*=.995).

The cluster-based permutation analysis yielded one positive cluster with a probability below the threshold (*p*-cluster = 0.002). For the time window generated by SHERPA, an effect of stimulus type is indicated at the electrodes that are highlighted in Fig. 5, respectively.

## Discussion

We developed an XAI-based pipeline that can identify EEG components from preprocessed epochs, without relying on null hypothesis significance testing statistics or prior assumptions. To validate our approach, we used a conventional face processing paradigm, which demonstrated the feasibility of an assumption-free method for analyzing EEG data. As hypothesized, we found the same ERP components in all three conditions of the study using our pipeline, which were consistent with those detected by the researcher as well as the data-driven approach. Like the other two approaches, SHERPA reliably identifies the N170 time window and sensor sites as being crucial indicators for face-related processing.

Further support for the feasibility of our approach comes from comparing it with cluster-based permutation. While there is considerable overlap between the electrodes identified as being crucial by both data-driven approaches, the SHAP values provide additional information. Posterior midline electrodes as well as some mid-frontal electrodes yield results in both approaches, however, SHERPA discards the later as rather unimportant and focuses more on the posterior electrodes. That is consistent with conventional analysis of early components which in a similar vein discards mid-frontal electrodes as they capture neural activity in the same cerebral regions as the posterior electrodes and thus often do not provide any additional information. In conclusion, the comparison with a conventional researcher-driven approach as well as well-established data-driven approach highlights the benefits of applying SHERPA to analyze electrophysiological data as it combines the strengths of both approaches. It neither relies on assumptions nor conventional (frequentists) statistics, but provides precise results that are intuitively interpretable.

### Reporting SHERPA results

SHAP values provide a way of decomposing the output of the model and attributing it to individual features, such as ERP components, in a mathematically sound and statistically rigorous way. This allows us to identify which ERP components are most important in predicting a particular outcome variable, such as behavioral performance, and to quantify the contribution of each component to the model’s prediction. The output of the classification model that is fed into the explainer is a probability distribution with values from 0 to 1 that add up to 1. Hence, the SHERPA “importance score” represent the contribution of each feature to the difference between the predicted probability and the expected probability given all the other features. The score thus depends on the units of the classification model‘s output. As these units are probabilities, SHERPA‘s output can be interpreted along the same line (Lundberg & Lee, 2017).

The SHAP scale represents the magnitude and direction of the contribution of a feature, such as an ERP component, to the output of a machine learning model. For our SHERPA approach, we used absolute values, with zero representing a neutral contribution, meaning that the feature has no effect on the output of the model. For example, a low or zero value for the face condition means that the underlying mechanism of the ERP is not related to face processing. A high SHERPA value for an ERP component indicates that the component has a strong positive contribution to the predictive outcome of the model, meaning that it is a critical component for predicting the outcome variable (again, face processing). Most importantly, the output scale is linear. For example, when comparing two SHERPA peaks and one is twice as high as the other, that means it is twice as important. If the peak is in conjunction with an ERP, this means that for the neural mechanisms, which is being investigated, that particular component is twice as important as well. Hence, SHERPA allows to directly assess whether a certain component indexes a neural mechanism and to which degree.

We suggest presenting the SHERPA results in their original form, as they are intuitively understandable; for example: “We found that the SHERPA scores for the [ERP component] were higher in [Condition A] compared to [Condition B] (SHAP score in [Condition A] = [value], SHAP score in [Condition B] = [value]). This indicates that [cognitive or behavioral measures] is more pronounced in [Condition A] as opposed to [Condition B].” When needed, researchers should provide any necessary additional information about the ERP component, including its latency, polarity, topography and its assumed relationship to cognitive or behavioral measures.

### SHERPA interpretation

Aside from the feasibility of our approach, this study provides first evidence for the new capabilities of XAI-based data analysis. SHERPA highlights the relevance of later time frames for the processing of facial features which hints not only at functionally distinct processes at early and later stages, but also indicates that the N170 other than thought might index a negative selection process.

As aforementioned, in all three conditions the SHAP values peak around the P100 and N170 as the literature would suggest. Scrambled and blurred stimuli show a sharp peak around 150ms. For faces, there are three peaks slightly different in amplitude between 150 and 300, but not as high in amplitude as for blurred or scrambled stimuli. Most importantly, for the face condition we also found a later, equally or even slightly more important component at around 300ms. Most interestingly, the lowest SHAP values for the face condition as opposed to the other conditions in the N170 component time window suggest that the processes occurring in this latency are not devoted to face processing per se. At those early stages of face processing, the N170 thus might indicate a negative selection process classifying the presented stimulus as *not* a face, and by that gate subsequent processing (Holmes, Vuilleumier, & Eimer, 2003).

The results are also in line with the interpretation from the original study stating that the N170 might bear less relevance for face processing (i.e., semantic processing) than later component (Recio, Sommer, & Schacht, 2011; Herbert, Sfärlea, & Blumenthal, 2013; Bublatzky, Gerdes, White, Riemer, & Alpers, 2014; Stolz, Endres, & Mueller, 2019; Ratcliff, Philiastides, & Sajda, 2009; Zion-Golumbic & Bentin, 2007). Conversely, if the stimulus presents a face, more resources are allocated to process it thoroughly as subsequently increased SHAP values suggest. The original study suggests that at these time window in-depth processing of the presented faces, i.e., consulting self-referential information and recognition of familiar faces takes place (Sagehorn et al., 2023). However, this study employed a virtual reality paradigm to get to the bottom of real-face paradigm, based on the premise that a 2D presentation of faces does not fully capture the processes associated with a real-life, i.e., 3D presentation in spatial proximity of the observer. Specifically, the premise of the study was that the 2D presentation obscures the full complexity of face processing by presenting low dimensional stimuli that do not conform to realistic setting and thus reduces the generalizability of conventional data. SHERPA likewise points in a similar direction.

Employing SHAP values as an indicator of importance enhances the sensitivity for detecting distinct processes. It is most curious that in any of the two other conventional approaches, the analysis of the later peak at around 300ms would yield a different interpretation. All three conditions have similar ERPs, with the blurred condition having the descriptively largest one. Previous approaches based on researcher or data-driven interpretations may not have concluded that different neural mechanisms are engaged at that time window. SHERPA goes beyond the microvolt values and provides evidence for functionally distinct processes associated with the same ERP waveform in the same experimental paradigm. This highlights the potential of SHERPA as a powerful tool for gaining insights into the neural mechanisms underlying cognitive processes.

### Limitations

While we successfully build a pipeline using an CNN and XAI capable of identifying important temporal and spatial clusters for EEG analysis, which were corroborated by expert knowledge and a well-known data-driven analysis method, using this pipeline potentially comes with certain drawbacks discussed below. Most feature importance measures like SHAP are not adapted to work with time series data which creates two problems. Firstly, the explainers are only capable of explaining all time points for one feature, or all features for one point, but not both simultaneously, thereby concealing the bigger picture. Secondly, many feature importance measurements like SHAP are theoretically unable to cope with correlated features (Molnar, 2022). However, despite the heavily correlated spatial and temporal features, SHAP gradient explainer seems to have provided plausible explanations. Other studies also found evidence that SHAP works well on time series (Alsuradi et al., 2020; Theissler et al., 2022). In the case of EEG data, neighboring electrodes and neighboring time points should be highly correlated leading to clusters of most important electrodes as well as small continuous time windows. This is exactly what we found with plausible coordinates for both.

Another potential drawback of a machine learning approach, especially as it involves a deep neural network and SHAP, is the high computational cost of the computation. For the first part, access to a GPU is imperative, while the second part needs a sufficiently large CPU. The computational cost produced by SHAP could be decreased by aggregating features (i.e., time points into time windows). However, the major advantage of EEG analyses over other neuroscientific methods (e.g., fMRI) is their extremely high temporal resolution. Hence, decreasing the granularity might conflict with the overall goal and benefits of this analysis.

This cost is somewhat alleviated by the fact that the EEG datasets from experimental psychology are typically rather small. However, having less data makes it more difficult to get reliable and generalizable results using ML. While the high number of trials per person in these kinds of experiments aids the classification, few participants naturally makes generalizations across participants harder. Nevertheless, for very basic processes like face perception, differences between persons might be less relevant as they are naturally small.

## Conclusion

To the best of our knowledge SHERPA is the first attempt at identifying temporal and spatial patterns of importance in EEG data by means of an XAI-based analysis. Hence, this study serves as a pilot to assess the suitability of XAI methods to aid in the analysis of ERPs. We aimed to build a first straightforward approach which firstly can easily be compared to established methods and secondly be transferred to and used with other experiments.

SHERPA offers researchers a way to study EEG data beyond the confines of null-hypothesis testing, and thus overcome its limitations. Unlike methods that depend on clustering, SHERPA makes full use of the spatial distribution of EEG sensors and allows to determine the spatiotemporal location with statistical precision without the risk of overestimating the resulting effects. One of the key benefits of SHERPA is its ability to increase the sensitivity of the EEG signal by reporting SHAP values. These values indicate the relative importance of different neural processes and enable researchers to differentiate between them with greater precision. By offering a more nuanced understanding of the EEG signal, SHERPA may contribute to a better understanding of brain activity and advance our knowledge of neural processing.

### Open Practices Statement

The datasets presented in this study can be found in online repositories. The names of the repository/repositories and accession number(s) can be found at: https://osf.io/y8c6q/?view_only=0d2fd8d6bd1e4351afe0deb5e3f4d3a4. The experiment was not preregistered.

## Data Availability

The Python code for SHERPA including a working example is available on GitHub: https://github.com/sophiasylvester/sherpa

## Footnotes

1 https://www.tensorflow.org/

2 https://scikit-learn.org/stable/

3 https://shap.readthedocs.io/en/latest/

4 https://scipy.org/

5 https://www.biosemi.com/headcap.htm

## Declarations

### Disclaimer

SHERPA, our novel method for analyzing EEG/ERP data using SHAP values, is named after the Sherpa people of the Himalayas, who have a long history of guiding climbers to the world’s highest peaks. Our method is designed to provide researchers with a reliable and efficient tool for navigating the complex landscape of ERP data, much like the Sherpa approach to mountaineering. The name SHERPA is a homage to the Sherpa people’s deep understanding of their environment, their precise navigation skills, and their rich cultural heritage.
